# The complete chloroplast genome sequence of *Stellera chamaejasme* f*. chrysantha* (Thymelaeaceae)

**DOI:** 10.1080/23802359.2020.1810166

**Published:** 2020-09-01

**Authors:** Chengbo Liang, Jiuxiang Xie, Jingyan Yan

**Affiliations:** a College of Agriculture and Animal Husbandry, Qinghai University, Xining, China; b State Key Laboratory of Plateau Ecology and Agriculture, Qinghai University, Xining, China

**Keywords:** *Stellera chamaejasme* f. *chrysantha*, Thymelaeaceae, chloroplast, phylogenetic analysis

## Abstract

*Stellera chamaejasme* L. f. *chrysantha* S. C. Huang is a toxic perennial herb of Thymelaeaceae and has the potential for medicine as *Stellera chamaejasme* L. Here, we present the complete chloroplast genome sequence of *S. chamaejasme* f. *chrysantha* based on Illumina sequencing data. The complete chloroplast genome sequence is 173,364 bp in size and contains four subregions: a pair of inverted repeats (IRs, each for 41,978 bp), a large single-copy region (LSC, 86,558 bp), and a small single-copy region (SSC, 2,850 bp). 141 genes were recognized in the assembled sequence: 93 protein-coding genes (PCGs), 38 tRNAs, 8 rRNAs, and 2 pseudo genes. The phylogenetic analysis result strongly supported that *Stellera chamaejasme* f. *chrysantha* was closely related to *S. chamaejasme* L.

*Stellera chamaejasme* L. is a toxic perennial herb which belongs to Thymelaeaceae, a family of Malvales (Zhao and Kang 2019). *Stellera chamaejasme* L. has obvious features on morphology, such as cespitose and unbranched stems, a terminal head with an involucre and the articulated hypanthium (Narae Yun et al. 2019). *Stellera chamaejasme* L. has been widely used in traditional Chinese medicine (Zhang et al. 2016). *Stellera chamaejasme* f. *chrysantha* is a form of *S. chamaejasme* L. and can be distinguished from *S. chamaejasme* L. through its yellow flowers. Previous study reported that, in China, *S. chamaejasme* f. *chrysantha* could be found in Yunnan, Sichuan, and the Nei Monggol Autonomous Region (Zhao et al. 2000). It was imported by Royal Botanic Gardens, Edinburgh, UK, for the ornamental purpose (Jian et al. 2005) and had been used in traditional Chinese medicine, taking the place of *S. chamaejasme* L. (Zhao et al. 2000). We sequenced the complete chloroplast genome of *S. chamaejasme* f. *chrysantha* in order to provide useful genetic information for further study on genetic diversity of Thymelaeaceae species.

In this study, *S. chamaejasme* f. *chrysantha* were collected near Spruce Meadow, Yunnan, China (27°13′42′′N, 100°22′54′′E). The specimen was kept in the Key Laboratory of Landscape Plant Lab, College of Agriculture and Animal Husbandry, Qinghai University, Xining, China (accession number: YJY-2019-YJY043). The chloroplast genome was extracted from fresh leaves. After genomic DNA extraction, genomic sequencing was performed on the Illumina NovaSeq Platform (Illumina, San Diego, CA) with a read length of 150 bp. The software SPAdes v3.10.1 (Bankevich et al. 2012) was used to assemble the chloroplast genome. Then PCGs, rRNAs, and tRNAs were annotated by Prodigal v2.6.3, Hmmer v3.1b2, and Hmmer v3.1b2, respectively.

The complete chloroplast genome of *S. chamaejasme* f. *chrysantha* (GenBank accession is MT790358) which is 173,364 bp in length (the GC ratio is 41.34%) exhibits a general quadripartite structure. Two inverted repeats (IRs, each for 41,978 bp) divide the genome into two single-copy regions, SSC and LSC with 2,850 bp and 86,558 bp, respectively. 141 genes are recognized in the complete chloroplast genome: 93 PCGs, 38 tRNAs, 8 rRNAs, and 2 pseudo genes. 13 of them contain one intron and 2 of them contain two introns.

Based on the complete chloroplast genomes assembled here and downloaded from GenBank, phylogenetic relationships of 8 Thymelaeaceae species (2, 1, 1, 1, and 3 from Stellera, Daphne, Pimelea, Wikstroemia, and Aquilaria, respectively) with two species from Myrtaceae as outgroups were resolved by the mean of neighbor-joining (with 10,000 bootstrap repeats). After aligned using MAFFT 7 (Katoh and Standley 2013), the neighbor-joining tree (Figure 1) was built using MEGA7 (Kumar et al. 2016). Phylogenetic relationships indicated by the phylogenetic tree were in agreement with their relationship based on morphological characteristics.

**Figure 1.**
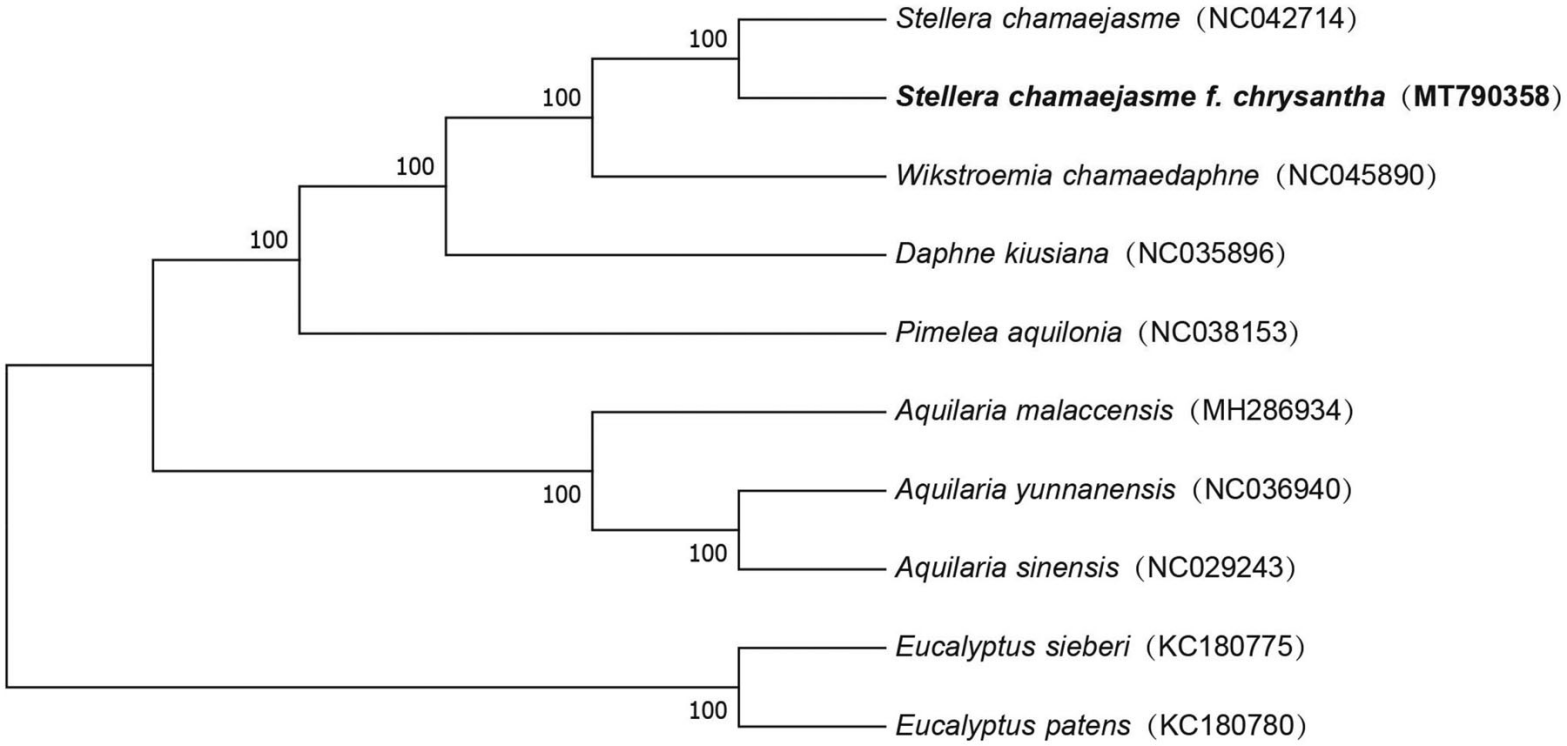
The Neighbor-joining phylogenetic tree based on 10 complete chloroplast genome sequences.

## Data Availability

The data that support the findings of this study are openly available in GenBank of National Center for Biotechnology Information at https://www.ncbi.nlm.nih.gov, reference number MT790358.
